# Infection Prevention Control Strategies of New Delhi Metallo-β-lactamase Producing *Klebsiella pneumoniae*

**DOI:** 10.3390/healthcare11182592

**Published:** 2023-09-20

**Authors:** Narcisa Muresu, Giovanna Deiana, Marco Dettori, Alessandra Palmieri, Maria Dolores Masia, Andrea Cossu, Cristina D’Avino, Illari Sechi, Arcadia Del Rio, Andrea Piana, Paolo Castiglia

**Affiliations:** 1 Department of Humanities and Social Sciences, University of Sassari, 07100 Sassari, Italy; nmuresu@uniss.it; 2 Medical Management, Hygiene, Epidemiology and Hospital Infection, University Hospital of Sassari, 07100 Sassari, Italy; madettori@uniss.it (M.D.); luca@uniss.it (A.P.); cristina.davino@aouss.it (C.D.); piana@uniss.it (A.P.); castigli@uniss.it (P.C.); 3 Department of Biomedical Sciences, University of Sassari, 07100 Sassari, Italy; delrio.arcadia2@gmail.com; 4 Department of Medicine, Surgery and Pharmacy, University of Sassari, 07100 Sassari, Italy; mdmasia@uniss.it (M.D.M.); andreacossu@uniss.it (A.C.); illasechi@uniss.it (I.S.); 5 Department of Restorative, Pediatric and Preventive Dentistry, University of Bern, 3012 Bern, Switzerland

**Keywords:** New Delhi metallo-β-lactamase, *Klebsiella pneumoniae*, multi-drug resistance, infection prevention control

## Abstract

The spread of multi-drug resistant organisms (MDROs) is increasing at an alarming rate worldwide. Among these, Carbapenemase-producing New Delhi Metallo-β-lactamase (NDM) poses a significant clinical threat, and appropriate measures must be taken to prevent or limit its penetration into still-free territories. The present report describes two independent cases of patients from Ukraine colonized by NDM-producing *Klebsiella pneumoniae* and admitted to two separate wards of an acute university hospital in a territory not yet affected by Carbapenemase producers of this class. Moreover, this report illustrates the infection prevention control (IPC) strategies promptly implemented by the IPC operational team to verify the possible spread of the microorganism in the ward and avoid any possible further contamination. The identification of genes coding for Carbapenemases, performed using real-time PCR, revealed no other cases within the wards involved. These cases emphasize the importance of early case recognition of multidrug-resistant bacteria, the necessity of effective inter-hospital communication, the need for effective antimicrobial stewardship protocol, and the importance of adequate IPC policies. Additionally, we highlight the need to improve screening procedures in the case of patients from countries with a high prevalence of MDRO, as essential measures to prevent potential nosocomial outbreaks and/or endemization.

## 1. Introduction

The spread of multi-drug resistant organisms (MDROs) is increasing at an alarming rate worldwide and represents one of the main threats for global public health, due to the high associated mortality and morbidity rates, prolonged days of hospitalization, the increased use of diagnostic and therapeutic procedures, and consequently, a significant increase in health care costs [1,2,3]. According to recent estimates, MDRO infections cause more than 50 thousand deaths between Europe and the United States each year, and more than 10 million deaths are expected by 2050 [4].

As recommended by the World Health Organization (WHO), a monitoring and control network for antimicrobial resistance (AMR) is essential to contain and prevent the spread of MDROs through active surveillance protocols, the prevention of hospital-acquired infections (HAIs), and the appropriate use of antibiotics in clinical and veterinarian settings [5].

During the last decade, the alarming emergence of Carbapenemase-producing New Delhi Metallo-β-lactamase (*bla*NDM) among Gram-negative bacteria posed a significant clinical menace due to the rapid spread of extremely drug-resistant clones [6]. NDM Carbapenemase was first reported in India in 2008, and since then, it has cropped up in various parts of the world, highlighting its speedy and easy dissemination across borders [7,8].

In 2018, a large and persistent outbreak caused by a single clone of *bla*NDM-positive carbapenem-resistant Enterobacteriaceae (CRE) was reported in Tuscany, Italy. Although the Tuscany region had an effective AMR screening program, the identification of the outbreak was delayed, resulting in a considerable increase in new cases and associated mortality [9,10]. In this scenario, the identification of individuals at high risk of carbapenemase carriage and the adoption of the most appropriate screening strategies are the key to reducing the burden of AMR.

Here, we reported a reliable and effective management of the first two independent cases of patients colonized by NDM-producing *Klebsiella pneumoniae*, admitted to two physically separated wards of an acute university hospital, based on the screening of contacts and the rigorous application of standard preventive measures.

## 2. Case Report

The present study did not require ethical approval for its observational design according to Italian law (Gazzetta Ufficiale n. 76 dated 31 March 2008).

### 2.1. Setting

The two cases occurred in the University Hospital of Sassari in Sardinia, Italy, the main hospital in the Italian region in terms of the number (861 beds) and diversity of its technological and professional resources, which provides services for numerous acute care clinical specialties. A CPE/CRE surveillance and control protocol has been activated since 2016 in the most critical wards at hospital admission, in order to provide clear indications regarding the prevention, early identification, and management of cases of patients colonized by MDROs [11]. The active screening protocol has been extended over time to all hospital wards and counts about 1000 rectal swabs analyzed monthly, with an average positivity rate of slightly below 2%. Although local epidemiology is overwhelmed by the wide circulation of two major clones (i.e., *Klebsiella pneumoniae* producing harboring *bla*KPC and co-harboring *bla*KPC and *bla*OXA-48 like), a steady increase in the number of species and Carbapenemase variants involved was registered over time, sometimes requiring prompt and enhanced preventive actions [12,13].

Routine screening for the microbiological identification and characterization of carbapenemase-producing Enterobacteriaceae (CPE) includes the collection and the delivery of rectal swabs to the hygiene laboratory. Subsequently, samples are cultured on Chromid Carba Smart Agar (bioMérieux, Grassina, Italy) [14]. After overnight incubation, suspect colonies are further investigated for identification using the Vitek-2 System (bioMérieux, Marcy l’Etoile, France) and, simultaneously, multiplex real-time PCR analysis is carried out to characterize the genetic determinants of AMR among the most common β-lactamases genes, *bla*KPC, *bla*NDM, *bla*VIM, *bla*OXA-48, and *bla*IMP, using the commercial kit Allplex Entero-DR assay [15,16].

Microbiological identification is followed by antimicrobial susceptibility testing, using the automated system Vitek-2 and the N379 card which provide the susceptibility profile for the following antibiotics: Amoxicillin/Clavulanate, Piperacillin/Tazobactam, Cefepime, Cefotaxime, Ceftazidime, Ceftolozane/Tazobactam, Meropenem, Imipenem, Amikacin, Gentamicin, Tobramycin, Ciprofloxacin, Trimethoprim/Sulfamethoxazole, and Ceftazidime/Avibactam. Drug susceptibility tests and minimum inhibitory concentration (MIC) values were assessed using breakpoints of the European Committee on Antimicrobial Susceptibility Testing (EUCAST) [17].

The hospital has a multidisciplinary team responsible for the prevention, surveillance, and control of HAIs and the responsible use of antimicrobials, the Infection Prevention and Control (IPC) team. In particular, the team deals with the surveillance of sentinel pathogens; the fight against antimicrobial resistance, both through the monitoring of the use of antimicrobial drugs and with the promotion of antimicrobial stewardship policies; the epidemiological surveillance of HAIs; the promotion of standard and additional precautions, with particular attention to hand hygiene; and professional training.

### 2.2. Clinical Details

The first case was a 63-year-old female inpatient of Ukrainian origin admitted to the geriatrics ward for dyspnea and fever in interstitial pneumonitis on 23 May 2021. The patient had recently been hospitalized for SARS-CoV-2 infection in Ukraine (until 7 May), where she underwent oxygen and antibiotic therapy. The first swab taken on admission was negative. According to the protocol, after 48 h, the second swab was analyzed, resulting positive for NDM-producer *Klebsiella pneumoniae*. The patient was hospitalized for 9 days.

The second case was a 73-year-old female inpatient of Ukrainian origin admitted in the urology ward for a secondary neoplasm of the right kidney on 2 December 2022. Based on current protocols, a rectal swab was taken on admission and was positive for the presence of CRE. After nephrectomy surgery, the patient was discharged on 10 December in overall good clinical condition.

The molecular analysis revealed the presence of NDM-producing *Klebsiella pneumoniae* in both specimens, with a marked resistance profile for all classes of antibiotics (Table 1).

### 2.3. Case Management

In cases where CPE is detected, based on the current protocol, immediate telephone notification is given to the ward. Afterward, a communication is sent via e-mail to the members of the IPC team, who are responsible for monitoring that all containment and isolation measures are in place. The patient is informed about their status of being a carrier and made aware of taking proper preventive measures. The patient’s general practitioner is also informed via letter upon discharge.

Rigorous IPC measures were introduced according to international guidelines, which foresee the immediate disposition of patients into a single room with a dedicated toilet and shower facility [18,19]. Strict contact precautions were put in place with gloves and gowns worn by all staff and visitors entering the room. Hand hygiene practice, according to WHO indications [20], was reinforced. The extra cleaning and disinfection of frequently touched surfaces with a sodium hypochlorite solution was introduced in the isolation rooms.

Following the approved protocol for CPE/CRE, an active screening program, addressed to all contact cases (i.e., inpatients attended to by the same healthcare team and within the same ward), was promptly started to identify other potential NDM carriers among patients. In both cases, we proceeded to collect rectal swabs from all inpatients, three times on alternating days, to confirm the first negative outcomes. A total of 73 swabs were analyzed for the first case and 42 for the second. Additionally, for the second case, we decided to perform molecular analysis directly on the rectal swabs. All samples were subjected initially to DNA extraction and later tested using real-time PCR. The screening activities did not reveal any further cases of patients colonized/infected by NDM-producing *Klebsiella pneumoniae*. To date, no other rectal swabs tested for the screening program have resulted positive for NDM-producing *Klebsiella pneumoniae*.

## 3. Discussion

The present report describes two independent cases of patients from Ukraine colonized by NDM-producing *Klebsiella pneumoniae* and admitted to two separate wards of an acute university hospital in a territory not yet affected by Carbapenemase producers of this class. Moreover, the report illustrates the infection prevention control (IPC) strategies promptly implemented by the IPC operational team in order to verify the possible spread of the microorganism in the ward and avoid any possible further contamination. It is important to underline that the cases reported were within wards not usually classified as “high-risk” wards. However, the possible use of invasive procedures or devices and the presence of vulnerable individuals, due to age and comorbidities, increased the risk of colonization and infection cases in urology and geriatrics wards. In this regard, a recent Italian study showed a statistically significant difference in *Klebsiella pneumoniae* prevalence between “high-risk” vs. “low-risk” wards (5.6% vs. 2.0%), as well as a higher risk of progression from colonization to infection for patients hospitalized in “high-risk” settings (18% vs. 3.5%) [21].

In recent years, the phenomenon of AMR has increased significantly and constitutes one of the major public health problems worldwide, with significant implications both from a clinical point of view (increased morbidity, mortality, days of hospitalization, possibility of development of complications, possibility of epidemics), and in terms of economic fallout due to the additional cost required for the use of more expensive drugs and procedures, longer hospital stays, and possible disability [22,23,24]. Several recommendations and coordinated strategies to curb the phenomenon, recognizing AMR as a priority in health care, have been proposed by international bodies [25,26,27]. The problem can be traced back to different causes, such as the increased (sometimes inappropriate) use of these drugs in both human and veterinary medicine, the use of antibiotics in animal husbandry and agriculture, the spread of HAIs caused by antibiotic-resistant microorganisms, and an increased prevalence of resistant strains due to increased commuting and international travel [28,29,30,31]. In particular, the interaction between antibiotic therapy and the development of AMR is a complex and concerning phenomenon that has significant implications for public health, as antibiotic misuse and overuse can increase the likelihood of selective pressure, the acquisition and spread of resistance genes, and the persistence of resistant strains [22,28]. In Italy, overall antibiotic consumption is higher than in many European countries and is characterized by the wide use of broad-spectrum molecules, which have a higher impact on antibiotic resistance. In fact, in recent years, the use of drugs to treat infections caused by MDROs has increased by 60%, accounting for nearly 29% of hospital consumption [32].

Therefore, the importance of identifying and isolating MDRO-colonized patients early is a significant challenge for health systems worldwide, which face the spread of increasingly difficult-to-treat infections, particularly in hospitals and healthcare facilities. Indeed, conventional antibiotic-based treatments may not be effective against these infections, reducing available treatment options and making disease control more complex [33,34,35]. As a matter of fact, the present case reports emphasizes the need for the early recognition and laboratory detection of MDRO, as well as the relevance of an adequate IPC program, and the importance of implementing strict control measures. In an environment with resistant bacteria, specific antimicrobial stewardship measures must be taken during the complete hospitalization period of patients vulnerable to HAIs. Noteworthy is the need to keep the patient in isolation until the colonization proves negative. In fact, following these cases, special infection control measures were taken in our hospital, such as supervising hand hygiene compliance, environmental cleaning, and antimicrobial stewardship [36,37].

However, a proactive and timely approach can enable targeted preventive action to reduce the spread of these pathogens and limit the occurrence of nosocomial infections [38,39]. The early detection of MDRO-colonized patients requires an approach based on effective screening methods, which, according to WHO guidelines, should be tailored to the inpatient ward, the patient’s susceptibility, and the potential risk the patient poses to other inpatients [40]. Laboratory tests, such as microbiological culture and PCR, make it possible to define the local epidemiology of CRE, detect the presence of specific microorganisms, and determine their sensitivity to antibiotics [41,42]. In particular, our cases emphasized the use of two different methods of antimicrobial resistance gene detection (i.e., cultural and molecular analysis). Despite the extensive ongoing debate about the best option among molecular and cultural methods in CRE screening, we reported high reliability for both methodologies. However, it is important to highlight the significant time savings achieved through nucleic acid amplification tests that ensure reliable and prompt results and, consequently, better management of potential carriers within the hospital ward [43,44].

Increased international mobility, whether for socio-political, demographic–economic, environmental, or recreational reasons, has resulted in a significant increase in the circulation and transmission risk of MDROs, particularly when areas with a high incidence of antimicrobial resistance, such as Southeast Asia and India, are involved [45,46,47]. Estimates indicate that about 20–30% of international travelers are colonized by antibiotic-resistant microorganisms, including strains of bacteria such as methicillin-resistant *Staphylococcus aureus* and antibiotic-resistant *Escherichia coli* [48,49]. These microorganisms, once introduced into communities, can adapt and spread rapidly, endangering public health and making it more difficult to treat infections. As a matter of fact, we highlight the need to improve screening procedures in the case of patients from countries with a high prevalence of MDRO, as essential measures to prevent potential nosocomial outbreaks and/or endemization. Indeed, the increasing number of international travelers/refugees has highlighted the need to increase the preparedness of healthcare workers in the handling of foreign patients [50,51].

In addition, the exposure to foreign healthcare systems, particularly in those who have undergone surgery, can increase the risk of acquisition and is a major source of importation of resistant bacteria [52,53]. That is why, when one is admitted to a hospital or clinic, it is advisable to inform the medical staff of any recent stay in a hospital facility in general, especially if abroad. In our case, both patients had been admitted, in the months before admission to our care facility, to a hospital in Ukraine where, due to the presence of the war, the health system has suffered serious damage. In this regard, communication between the laboratory and clinicians regarding risk factors, such as travel history and previous hospital admissions, for patients with CRE is essential in identifying NDM producers and other new resistance phenotypes.

## 4. Conclusions

To the best of the authors’ knowledge, these are the first cases of *bla*NDM carriers found in the territory of Sardinia. Therefore, considering how, in the past, the first CRE strains spread so rapidly following penetration, and given the insularity of the region, it is even more important to maintain a high level of control in this area, which is currently considered free of these genotypes. The control of infectious risk and AMR is an objective that must be actively pursued. However, in order to be effective, it requires the multifactorial contribution of various actors: on the one hand, healthcare institutions must put in place the adequate governance and management of clinical risk and infectious risk (for example, stewardship programs, risk management activities, and sanitization procedures); on the other, it is essential to work on the sense of responsibility and awareness of professionals, health and social–health workers, and the community.

Addressing this problem is an imperative need, not only to improve individual patient care but also to protect public health and limit the spread of antibiotic resistance. In addition, there must be an investment in research to develop new drugs and strategies to combat AMR and identify more effective screening strategies and improve the management of colonized patients. A not insignificant aspect of AMR surveillance is the molecular characterization of isolated strains, aimed at monitoring circulating strains and the early identification of new clones. Thus, an advancement in current methodologies would be desirable to improve the effectiveness of surveillance protocols and initiate timely IPC measures to limit the spread of MDROs, among which are patient isolation, the use of targeted antibiotics, strict hand hygiene, and surface disinfection.

## Figures and Tables

**Table 1:** Antimicrobial susceptibility testing of NMD-producing *Klebsiella pneumoniae* strains.

Antibiotics	Case 1		Case 2	
	M.I.C.	S/R	M.I.C.	S/R
Cefepime	≥32	R	≥32	R
Cefotaxime	≥64	R	≥64	R
Ceftazidime	≥64	R	≥64	R
Ceftolozane/tazobactam	≥32	R	≥32	R
Meropenem	≥16	R	≥16	R
Imipenem	≥16	R	≥16	R
Amikacin	32	R	4	S
Amoxicillin/clavulanate	≥32	R	≥32	R
Piperacillin/tazobactam	≥128	R	≥128	R
Gentamicin	≥16	R	≤1	S
Tobramycin	≥16	R	≥16	R
Ciprofloxacin	≥4	R	≥4	R
Trimethoprim/sulfamethoxazole	≥320	R	≥160	R
Ceftazidime/avibactam	≥16	R	≥16	R

S = sensible; R = resistant.

## Data Availability

Data are available on reasonable request.
